# Pupil Size Tracks Attentional Performance In Attention-Deficit/Hyperactivity Disorder

**DOI:** 10.1038/s41598-017-08246-w

**Published:** 2017-08-15

**Authors:** G. Wainstein, D. Rojas-Líbano, N. A. Crossley, X. Carrasco, F. Aboitiz, T. Ossandón

**Affiliations:** 1 Departamento de Psiquiatría, Escuela de Medicina and Centro Interdisciplinario de Neurociencia, Pontificia Universidad Católica de Chile, Santiago, Chile; 2 Laboratorio de Neurociencia Cognitiva y Social, Facultad de Psicología, Universidad Diego Portales, Santiago, Chile; 3 Department of Psychosis Studies, Institute of Psychiatry, Psychology and Neuroscience. King’s College London, London, UK; 4 Servicio de Neurología y Psiquiatría, Hospital de Niños Dr. Luis Calvo Mackenna, Facultad de Medicina, Universidad de Chile, Santiago, Chile

## Abstract

Attention-deficit/hyperactivity disorder (ADHD) diagnosis is based on reported symptoms, which carries the potential risk of over- or under-diagnosis. A biological marker that helps to objectively define the disorder, providing information about its pathophysiology, is needed. A promising marker of cognitive states in humans is pupil size, which reflects the activity of an ‘arousal’ network, related to the norepinephrine system. We monitored pupil size from ADHD and control subjects, during a visuo-spatial working memory task. A sub group of ADHD children performed the task twice, with and without methylphenidate, a norepinephrine–dopamine reuptake inhibitor. Off-medication patients showed a decreased pupil diameter during the task. This difference was no longer present when patients were on-medication. Pupil size correlated with the subjects’ performance and reaction time variability, two vastly studied indicators of attention. Furthermore, this effect was modulated by medication. Through pupil size, we provide evidence of an involvement of the noradrenergic system during an attentional task. Our results suggest that pupil size could serve as a biomarker in ADHD.

## Introduction

Attention-deficit/hyperactivity disorder (ADHD) is the most prevalent childhood neuropsychiatric disorder^1, 2^. This condition is characterized by inattention, impulsiveness and hyperactivity, and is becoming increasingly recognized that many patients continue having these types of difficulties in adulthood^3^. Most of ADHD symptoms are related to problems in behavioral and cognitive control, and have been attributed to a deficient dopaminergic signaling^2, 4, 5^. First-line treatment for ADHD is given by stimulants, mainly methylphenidate, and the usual second-line treatment is atomoxetine, a noradrenaline reuptake inhibitor^2, 6^. Both kinds of drugs increase the catecholamine availability at synapses. This, together with evidence of a weak but consistent link between genetic polymorphisms associated to the catecholaminergic system and ADHD, prompted the widely accepted hypothesis that in this condition there is an underlying deficit in catecholaminergic neurotransmission^1, 7^.

Currently, diagnosis is being performed solely on the basis of observed behavior and reported symptoms, which carries the potential risk of over-diagnosis or under-diagnosis^6, 8^. Like any complex disorder, ADHD patients display clear heterogeneities at the clinical and biological levels. These differences probably explain why some patients do not respond to medication^6–9^. A biological marker that helps to objectively define the disorder, providing information about its pathophysiology and potential responses to medication, is needed^9, 10^.

A promising potential marker of cognitive states in humans is pupil size^11–15^. After controlling for stimulus luminance, pupil size correlates with task difficulty, emotional valence, physical effort, motor output and arousal states^11, 16–21^. Independent findings suggest that these fluctuations in pupil size reflect the state of the brain norepinephrine (NE) system^11, 22–26^. This system originates in the locus-coeruleus (LC) and projects throughout the cerebral cortex, hippocampus, thalamus and midbrain, among others^22–24, 27–29^. Brain areas associated with attentional processing (e.g., parietal cortex, pulvinar nucleus, superior colliculus) receive particularly dense LC-NE innervations^12, 25^. Data from animal models, both rodents and non-human primates, have shown a central role for the LC in selective attention^11, 23–25^.

Nowadays, pupil size can be monitored in a completely noninvasive way in humans, using a remote camera and infrared light. We hypothesized that if pupil size reflects the activity of the LC-NE system, which is one of the important attentional systems in humans, then pupil size is a potential and unexplored marker for attentional states in ADHD. If this is the case, changes in pupil size during an attentional task should reflect the behavioral differences observed between ADHD patients and control subjects.

**Figure 1:**
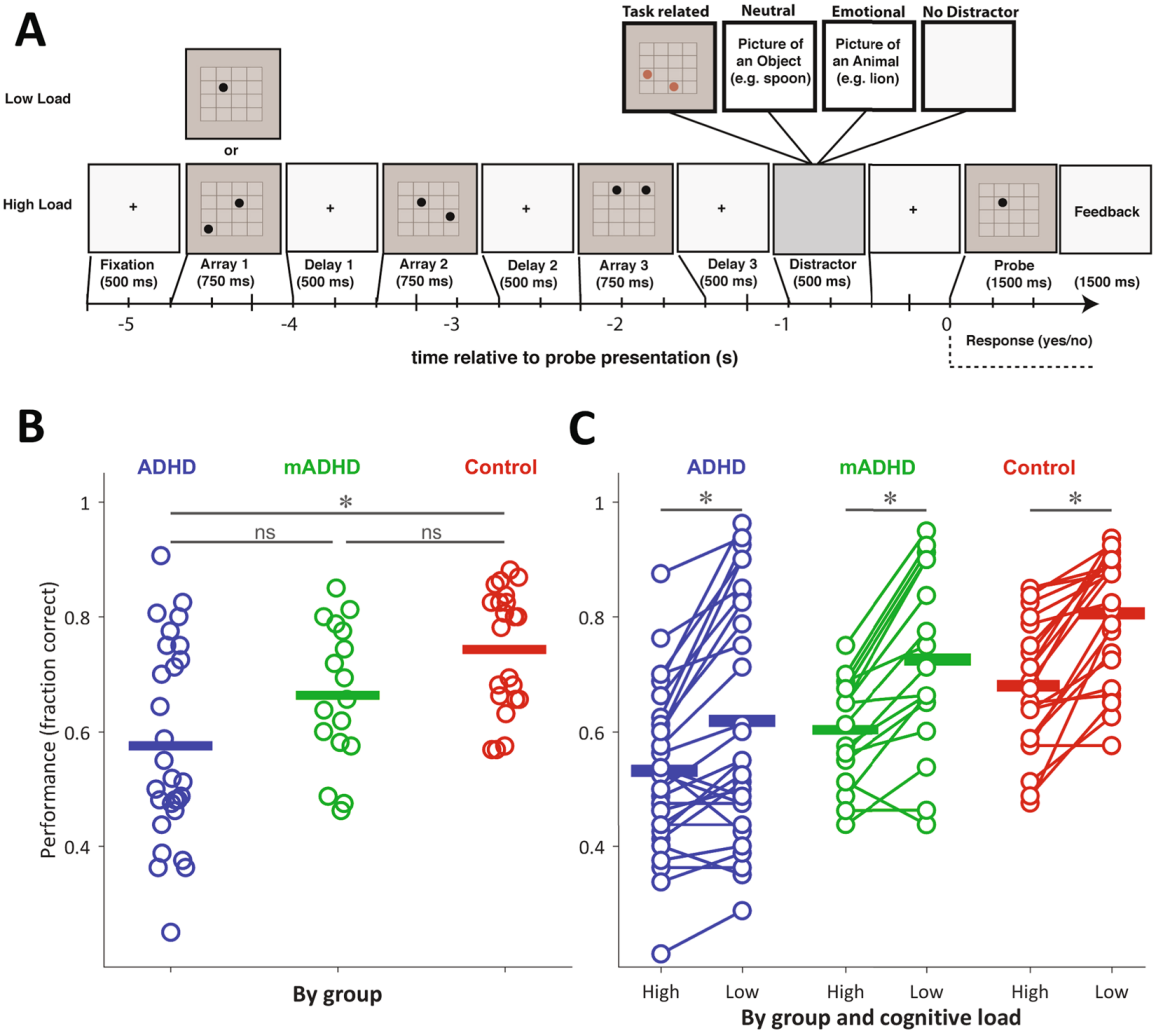
(**A**) Schematic representation of a single trial. Following the presentation of the probe, subjects indicated with a button press whether the position of the dot had been presented in one of the previous arrays. **(B)** Task performance by group. Each dot corresponds to the mean session performance of a subject. Horizontal bars correspond to the group mean. The on-medication ADHD group (mADHD) is a subset of the ADHD group, tested on a different day. **(C)** Performance by group and cognitive load. Each dot corresponds to the mean session performance of a subject, in high and low cognitive load conditions. In both (**B**) and (**C**), performance is expressed as the fraction of correct trials of the session. Differences at p < 0.05 are highlighted.

We monitored pupil size from ADHD and control subjects during a visuo-spatial working memory task (see Fig. 1A). Deficits in these tasks are one of the most consistent impairments of executive functions in patients with ADHD^30, 31^. Indeed, unaffected siblings present deficits in this type of task, which are intermediate between ADHD and controls^31^, a finding that has led some authors to propose it as an endophenotype^2, 32^. In our task, three images plus a distractor were sequentially presented and then a ‘probe’ image was shown. Subjects had to evaluate if the probe image had been presented before. ADHD patients performed the task in two separate sessions, on- and off-medication, which in all cases was methylphenidate. We found that pupil size changed during the probe presentation, the attentionally most relevant cue of each trial, and covaried with the subject’s variability in response time and performance. Furthermore, this effect was modulated by medication.

## Results

As a first step, we performed the analysis using all the data (28 ADHD subjects, 22 controls, and a subgroup (17) of the 28 ADHD children who performed the task twice, on-medication and off-medication). Here we described the data and compared between groups, both the behavioral and the physiological measures. As a second step, we specifically focused on the subset of subjects which performed the task on- and off-medication. In this way, we were able to assess for medication-associated changes on the same subject.

### Task performance results

To assess for performance differences between groups, we first conducted a Kruskal-Wallis analysis of variance, which gave significant differences (χ^2^_(d.f.=2)_ = 13.4, p = 0.0012). Post-hoc test performed through the Bonferroni method, showed differences only between ADHD and Control groups (ADHD vs. mADHD: p = 0.4018; mADHD vs. Control: p = 0.2146; ADHD vs. Control: p = 0.0008). We obtained the same differences via Tukey’s Honestly Significant Difference Procedure. When trials were parsed by cognitive load (low: 1-dot memoranda, high: 2-dot memoranda), the data confirmed that 2-dot arrays were harder for the subjects, across all groups. There were significant differences in performance levels for low and high cognitive loads, as assessed by a Wilcoxon signed-rank test (Control: high 0.68 ± 0.11, low 0.81 ± 0.11, z = 4.02; p < 0.0001; ADHD: high 0.53 ± 0.14, low 0.62 ± 0.21, z = 3.5; p < 0.0001; mADHD: high 0.60 ± 0.09, low 0.73 ± 0.16, z = 3.31; p < 0.0001). The plot in Fig. 1C shows the change in performance for each subject, related to low and high cognitive loads.

### Pupil diameter changes during the trial

**Figure 2:**
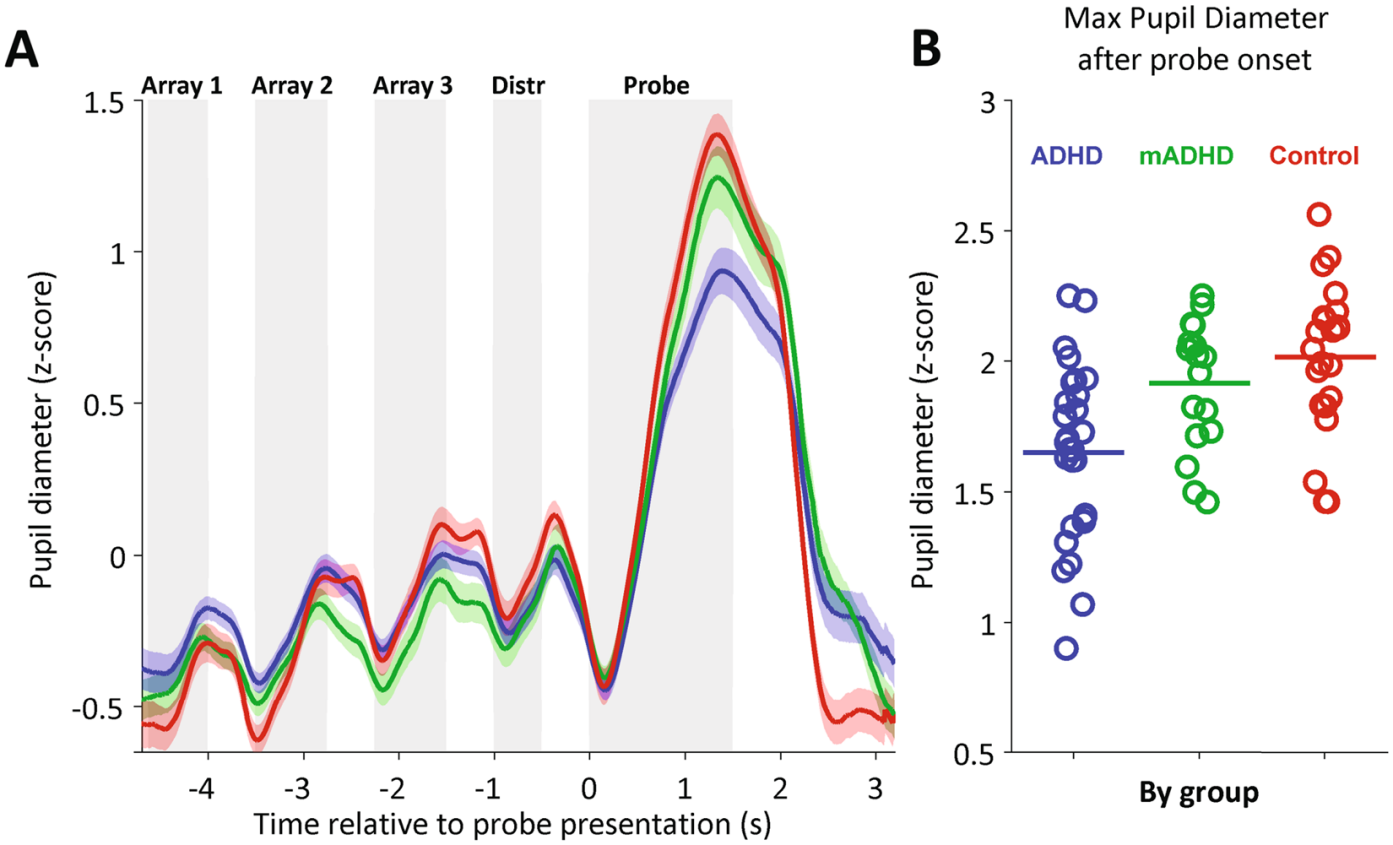
Mean pupil change during a trial and maximum pupil diameter after probe onset. **(A)** Mean pupil change during a trial, parsed by group. Each trace corresponds to the mean of each group (ADHD, n = 28; mADHD, n = 17; Control, n = 22). Color shaded areas correspond to the standard error of the mean. Grey shaded areas mark the periods of stimuli presentation during the trial. Dot Arrays 1–3: dot array presentations (See Fig. 1A); Distr: distractor image presentation; Probe: probe dot presentation. **(B)** Maximum pupil diameter measured after probe onset. Each dot represents the mean session value for a subject (i.e., average across trials). Horizontal bars correspond to session averages across subjects.

Average pupil curves across trials and subjects (Fig. 2A) reliably co-varied with visual stimuli presentation in the three groups. Each curve in Fig. 2A, which corresponds to an average across subjects, shows diameter increases associated with the presentation of the dot arrays, the distractor, and the probe dot. On average, the largest increase in pupil diameter in the three groups occurred after probe onset (to see individual pupil curves, see Supplementary Figure 1). To visualize and compare the actual maxima across trials, we performed a parallel analysis. Instead of looking at the average curve per subject, we used the same pupil timeseries to average the maximum pupil size for each subject and trial, after probe onset (see Methods). These values are shown in Fig. 2B. To assess for differences between groups in maximum pupil size after probe onset, we conducted a Kruskal-Wallis analysis of variance, which gave significant differences (χ^2^_(d.f.=2)_ = 15.57, p = 0.0004). Post-hoc tests conducted using the Bonferroni method showed significant differences between ADHD and mADHD, and also between ADHD and Control, but not between mADHD and Control groups (ADHD vs. mADHD: p = 0.0361; mADHD vs. Control: p = 1.00; ADHD vs. Control: p = 0.0004). We obtained the same differences via Tukey’s Honestly Significant Difference Procedure. As such, medication tended to “normalize” the pupil response of patients during the attentional task. Pupil diameter average magnitudes for the first three arrays presentations are shown in Supplementary Fig. 2.

### Associations between pupil diameter and behavioral variables

**Figure 3:**
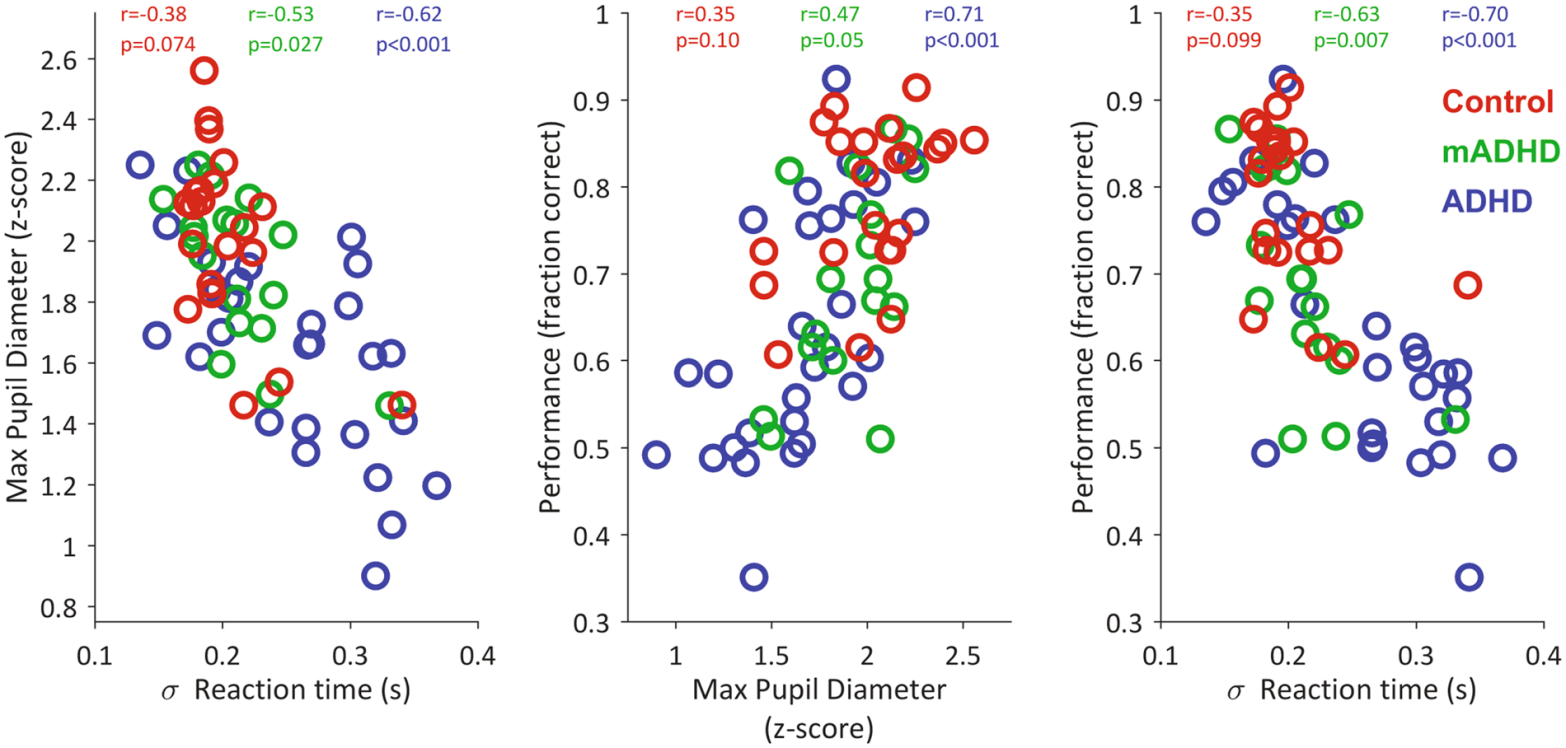
Relationships between task performance, reaction time variability and maximum pupil diameter after probe presentation. Each dot corresponds to the mean session value per subject. The values of the Spearman correlation coefficient (r: rho), separately by group, are shown at the top of each plot. Reaction time variability is presented as the standard deviation of the reaction time, which in turn corresponds to the time elapsed between probe presentation and subject’s response.

We then evaluated associations between pupil diameter and two of the most relevant behavioral variables: performance and variability in reaction time. In all the combinations, these variables showed consistent associations, as assessed statistically by the Spearman correlation coefficient (see Fig. 3). We used the subjects from all three conditions to analyze these associations. The maximum pupil diameter measured after probe presentation was inversely associated to the variability in the subject’s reaction time (Spearman correlation, rho = −0.68, p < 0.0001, n = 67). On the other hand, maximum pupil diameter was directly associated with the subjects’ performance in the task (rho = 0.63, p < 0.0001, n = 67), indicating that pupil size during the crucial part of the task was associated with the subjects’ outcome. Finally, consistent with previous reports and with the two previous associations, we found that performance was inversely associated with the variability in reaction time (rho = −0.725, p < 0.001, n = 67). In terms of correlations within groups, we consistently found that the largest correlation values corresponded to the ADHD group, intermediate values to the mADHD group, and lowest to the Control group (see rho values in plots of Fig. 3). Performance did not co-vary with raw reaction time (see Supplementary Figure 4). Furthermore, pupil diameter average magnitudes for the first three arrays presentations positively covariates with arrays presentation (see Supplementary Figure 2) in control (Spearman correlation, rho = 0.5864, p = 2.29858e-07, n = 66) and ADHD group (Spearman correlation, rho = 0.27962, p < 0.01, n = 84). Nevertheless, this increase in pupil diameter does not correlates with performance and pupil diameter measured after probe presentation within subjects (see Supplementary Figure 3).

### Paired comparisons between conditions on- and off-medication

For a total of 17 subjects, we were able to collect one off-medication session (children suspended their medication 24 hours before performing the task) and a matching on-medication session for the same subject. We used these data to evaluate differences between the on-medication and off-medication conditions for the experimental variables, both physiological (pupil diameter) and behavioral (performance, reaction time variability).

**Figure 4:**
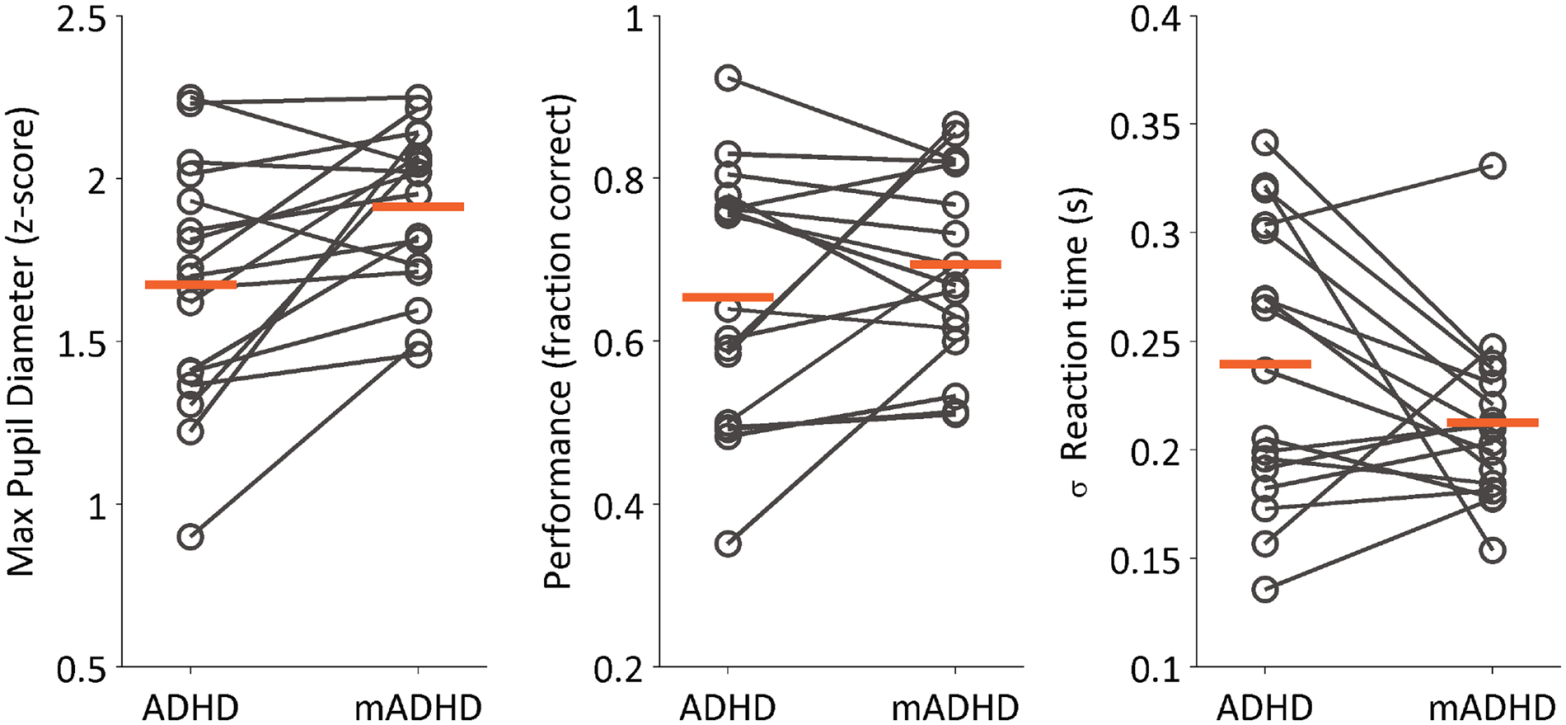
Paired changes between off-medicated (ADHD) and on-medicated (mADHD) conditions, for the subset of subjects which performed the task in both conditions (n = 17). Left: Change in maximum pupil diameter measured after probe dot presentation. Center: Change in performance, expressed as the fraction of correct trials of the session. Right: Standard deviation of the subject’s reaction time (time elapsed between probe presentation and subject’s response). In all plots, each dot represents the session mean for a subject. Orange horizontal bars correspond to the group mean.

During on-medication sessions, children displayed a larger pupil diameter during probe presentation compared to off-medication sessions, as assessed by a Wilcoxon signed-rank test (z = −2.95, p = 0.003, n = 17 subjects) (see Fig. 4). Performance, on the other hand, did not vary significantly between on- and off-medication conditions (z = −0.69, p = 0.4925, n = 17 subjects), neither did the standard deviation of the subjects’ reaction time (z = 1.54, p = 0.1239, n = 17 subjects).

### Medication-associated changes in pupil diameter and behavioral variables

**Figure 5:**
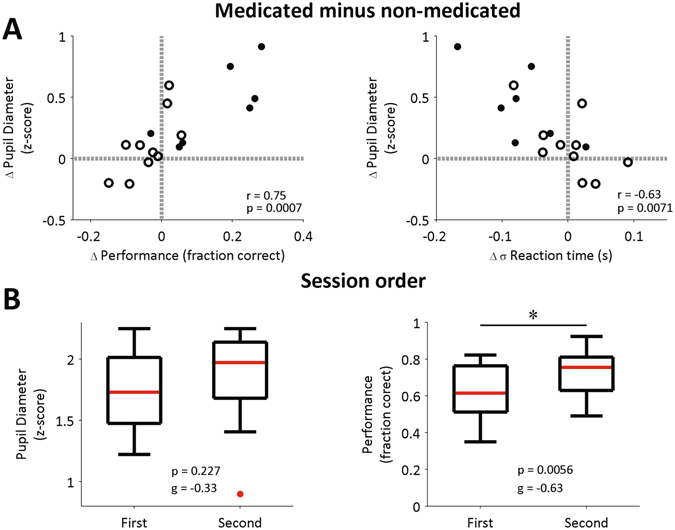
Medication-associated changes and session-order comparisons for ADHD subjects. (**A**) Association between change (delta, Δ) in pupil diameter after probe presentation, and changes in performance (left) and standard deviation of reaction time (right). The change is calculated as the mean difference between on-medicated and off-medicated conditions. Each dot represents the difference in session means for a subject. Grey reference lines are drawn at zero changes. Empty circles: subjects whose first session was on-medication; Filled circles: subjects whose first session was off-medication. r: Spearman’s rank correlation coefficient. p: p-value for testing the hypothesis of no correlation. (**B**) Pupil diameter (left) and performance (right) separately by session. Data were parsed by session, regardless of the medication condition (on- or off-). Each boxplot was built using the mean values from each of the 17 subjects. p: p-values from Wilcoxon signed-rank test. g: Hedges’ g (measure of effect size).

Finally, we analyzed the relations between pupil size and performance, and between pupil size and reaction time, specifically in the subset of subjects which performed the task in on- and off-medication conditions. To do this, we subtracted the mean variable values of the off-medication sessions from the mean values of on-medication sessions, separately for each subject. We added the prefix ‘delta’ (Δ) to the new variables representing the on-medicated minus off-medicated values. We then plotted these differences against each other (See Fig. 5A). For the case of Δ pupil diameter and Δ performance, we found a positive association, as measured by Spearman correlation (rho = 0.75, p = 0.0007). In the case of Δ pupil diameter and Δ reaction time variability, we also found an association (rho = −0.63, p = 0.0071). These results indicate that if the medication was accompanied by a large increase in performance (or a large decrease in reaction time variability) for a given subject, then it was also accompanied by a large change in pupil diameter. Interestingly, we noted that the subjects who displayed a larger change in performance were subjects which performed first the task off-medication (filled circles in Fig. 5A). We therefore next looked at mean values parsed by session, irrespective of medication condition (Fig. 5B). We found that there was a small but significant effect of session on performance (Wilcoxon signed-rank test, z = −2.76, p = 0.0056, Hedges’ g = −0.63, first minus second) and not on pupil size (Wilcoxon signed-rank test, z = −1.27, p = 0.227, Hedges’ g = −0.33, first minus second).

These results show that there could be an effect of medication on the observed pupil and reaction time changes, but in our data there is a potential confound with training, measured as session order.

## Discussion

We present evidence supporting pupil changes during an attentional task as a promising biomarker for ADHD. It has been recently shown in animal experiments that pupil diameter tracks arousal and attentional states^12, 23, 27^. Specifically, pupil diameter covaries with neuronal activity in the LC-NE system, the so-called ‘arousal’ or ‘alerting’ network. The LC-driven norepinephrine modulation, which reaches almost the entire cortical surface, has a direct readout in pupil size^23^. This confluence of evidence from NE involvement in attentional circuits and control of pupil size prompted us to explore pupil size as a biological marker in ADHD.

We tested ADHD and control children in a visuo-spatial working memory task (Fig. 1A) previously known to be challenging for ADHD patients. A subset of ADHD children performed the task both in on-medication and off-medication conditions. Off-medication ADHD patients showed decreased pupil diameter response during the task as compared to controls (see Fig. 2). On average, this difference was no longer present when patients were on-medication. Specifically, we saw in our task an event-related pupil response which tracks the array presentations and displays the largest increase during the probe dot presentation. When the probe dot was presented, subjects had to pay attention to it, compare it with what they recall about the previous three arrays and make a decision. In this crucial moment of the task, the size of the induced pupil response helped us to distinguish between patients and control subjects. Given that pupil diameter reflects the activity of the LC-NE system^11, 12, 22, 23^, these results support the involvement of the cerebral NE system in ADHD.

We found a correlation between pupil size and subjects’ performance, measured as response accuracy and reaction time variablility, in the ADHD group. These performance metrics, are two vastly studied behavioral ADHD markers in attentional tasks^9, 33, 34^. In this study, the correlations between these markers and pupil size were weaker in the on-medication group and display their lowest values in the control group. This association between pupil size and behavior seems to be restricted to the period after probe onset. For example, the sustained baseline increase across array presentations (see Fig. 2A) did not correlate with response accuracy or reaction time variability (see Supplementary Figures 2 and 3). It has been proposed that these manifestations of ADHD (increased reaction time variability and poor accuracy in attentional tasks) could be caused by transient disruptions of attentional network activity^33, 34^. These disruptions are in turn caused by an increase in the activity of other internal networks, such as the default mode network. In addition, recent models have proposed the role of LC-NE circuits in so-called ‘mind wandering’ periods, which might potentiate the default mode network and decrease the activity of attentional networks^35^. This interference is characterized by a decrease in the phasic gain of the LC-NE system which, in turn, causes decreased pupil diameters^35^. Thus, there are compelling reasons for using pupil-size dynamics to characterize and diagnose ADHD.

We also found that the subjects whose performance and reaction time variability changed more with the medication, also displayed larger pupil-size change between on-medicated and off-medicated conditions (Fig. 5). Hence, the co-variation between pupil size change and behavioral change seems subject-specific. We note that the session order (on-medication or off-medication) was randomized in our subjects, which means that some of them learned the task off-medication and then were tested a second time on-medication. Besides, performance, pupil diameter and reaction time variability did not consistently change across trial blocks (see Supplementary Figure 5). This suggests that motivational or learning effects were not modulating these responses.

Overall, the association between pupil diameter changes and ADHD behavioral performance markers, and how these associations differ between ADHD and normal children, suggest that pupil diameter changes during a visual-spatial working memory task may be a useful biological marker of ADHD. These associations are also consistent with the hypothesis that ADHD is a neurodevelopmental disorder related with the broadly distributed NE system, and not only a problem confined to a prefrontal-striatal dopamine circuit, as implied by the classical version of the dopaminergic hypothesis of ADHD^5, 36^.

## Methods

### Subjects

A total of 50 subjects participated in the study. 28 subjects were patients diagnosed with ADHD (4 girls, age: 10.71 ± 0.54 years old). All ADHD children were being treated with methylphenidate. 22 subjects were non-ADHD assigned to our control group (4 girls, age: 11.58 ± 0.50 years-old). A subgroup of 17 ADHD patients (3 girls, age: 11.19 ± 0.86 years-old) performed the task twice, on- and off-medication (time between sessions: 180.23 ± 125.17 days). The order of the sessions (on- or off-medication) was randomized to avoid carry-over effects. In the on-medication session, ADHD subjects took their regularly prescribed dose 2–2.5 hr before the task, which is the time corresponding to the maximum drug concentration after oral administration^37^. For off-medication sessions, children discontinued their medication 24 h prior to the day of testing, which is enough time to remove 80% of the circulating drug levels^38, 39^. No significant differences were found between the groups in age, IQ, or educational level. The ADHD group had a mean IQ of 100 (SD =  ± 11.6), while the non-ADHD group had a mean IQ of 105 (SD =  ± 7.3). All ADHD subjects were diagnosed as ADHD-Combined Subtype by a trained child neurologist according to the DSM-IV criteria^40^. All participants were also screened for neurologic or psychiatric comorbid conditions using a protocol that included parents’ interview, M.I.N.I. Kid test^41^, and general psychological and physical assessment of the children. The Conner’s Abbreviated Parent–Teacher Questionnaire^41^ is usually utilized to screen for symptoms of ADHD in the clinical setting. In the present study, it was used as an additional symptoms-counting tool. Participants with ADHD and those from the non-ADHD group were evaluated using this instrument as a controlling variable. Parents granted informed consent for their children’s participation, and children signed an informed assent form. The procedures in the study were approved by the Ethics Committee of the School of Medicine of the Pontificia Universidad Católica de Chile (Protocol number 11082), in accordance with the Declaration of Helsinski.

### Task

Subjects performed a Sternberg-type delayed visuo-spatial working memory task, adapted from Dolcos & McCarthy^42^. The memoranda were 1- or 2-dot arrays, with the dots located variably in any of the sixteen spaces of a 4 × 4 grid (See Fig. 1A).

On each trial, subjects were instructed to start by fixating on a black cross located at the center of the screen. After 500 ms, dot array presentation commenced. Three different dot arrays were presented on each trial. Each array was presented for 750 ms, with a 500 ms delay between arrays, during which a fixation cross was presented (see Fig. 1A). After the last delay period, a distractor image was presented for 500 ms. After the distractor, a ‘probe’ dot was presented for 1.5 seconds. This was a dot within the grid, and subjects had to answer ‘yes’ if the probe dot had been presented in one of the trial’s previous arrays, or ‘no’ if it had not. Immediately after probe offset we provided a feedback image for 1500 ms, indicating if the subject response was correct or incorrect. The participants were instructed to respond as fast as possible.

We used three distractors: (1) A task-related dot array; (2) a neutral natural image; and (3) an emotional image. Distractors were constructed and modified from public domain images, adapted from Vidal, Ossandon *et al*.^43^. In 25% of the trials, no distractor was presented. Trial types were presented randomly.

There were also two trial types, according to cognitive load: in low-load trials, only one dot was presented on each image, whereas in high-load trials, two dots were presented on each image. Therefore, in low-load trials subjects had to retain the location of three dots (one per image) and in high-load trials they had to retain the location of six dots (two per image).

A total of 160 trials were presented on each session, separated in 8 blocks of 20 trials. Sessions usually lasted 30 minutes.

### Data Acquisition

Pupil diameter data was acquired with Eyelink 1000 (SR Research Ltd., Mississauga, Ontario, Canada), with a 1 kHz sampling frequency. Subjects sat in front of a table containing the computer screen for image presentation and the eye tracker device. During the task, the subjects kept their head in a forehead/chin rest (SR Research Ltd.). Subjects were placed at a viewing distance of 30 cm from the display monitor. We measured illuminance data with a lux-meter positioned in the same chin-rest used by the subjects, at the same distance from the screen. The only modestly large differences in illuminance occurred between delay and array displays. The distractors presented more variability in illuminance and had lower illuminance values, on average (see Supplementary Figure 6). Illuminance changes were always on the order of 1 lux (as a reference, a change from white to black screens, in our setup, is associated with a drop of ~100 lux).

### Behavioral Data Analysis

Stimuli were presented using Presentation® software (Neurobehavioral Systems, Inc.), and subjects delivered their responses using a keypad. Alongside checking for correct responses, we also recorded the reaction time as the time delay between the probe presentation and the subject’s response. This allowed us to calculate the variability in reaction time, a parameter known to correlate with cognitive performance in ADHD children^44, 45^.

### Pupil Data Analysis

Pupil diameter analysis was performed using Matlab® software with in-house functions. First of all, periods of blinks, in which no pupil diameter information was available, were detected by the Eyelink software. Pupil data surrounding blinks were removed from the time series used in the analyses. Pupil diameter during these periods was estimated using a cubic spline interpolation^46^, implemented through Matlab function *spline*.

To obtain the pupil diameter average profile (Fig. 2A), data of each participant were baseline-adjusted and smoothed by a bandpass Butterworth filter between 0.025 Hz and 4 Hz. A low-pass frequency filter extracted the high frequency noise^29^, and a high-pass filter was applied to detrend the basal slow change of the pupil diameter across trials. Furthermore, outliers, defined as periods of pupil change (derivative function) higher than 3 standard errors from the mean were discarded. Finally, all trials with more than 50% of missing data (due to blinks or outliers) were not considered in the analysis. Unless otherwise stated, data analysis was restricted to the 8-s trial period (Fig. 1A). After filtering and outlier removal, the pupil timeseries was normalized by means of a z-score, separately for each trial.

To calculate the pupil diameter maxima during probe presentation (Fig. 2B), we used the same z-scored pupil time series used in Fig. 2A. We defined a window between 500 ms after probe onset and 500 ms after probe offset. Within that window, we calculated the maximum value of pupil diameter for each trial. We then computed the mean value of those maxima, separately per subject, to obtain each data point in Fig. 2B.

### Statistics

All our statistical tests were implemented in Matlab® (release 2016a). Due to the non-normal nature of the data, we used non-parametric tests to assess statistically for differences. For paired comparisons, we used the Wilcoxon signed-rank test, with Matlab function *signrank*. For non-paired comparisons, we implemented permutations tests using a t-statistic (difference between group means normalized by a pooled standard deviation). In this case, we first computed the t-statistic for the actual (non-permuted) data. We then implemented the permutations and calculated the t-statistic for each permutation. To assess significance, we compared the non-permuted t-value with the distribution of permuted t-values and calculated if it lied within 95% of the distribution (i.e., we used a significance level alpha = 0.05). All tests were performed using the subject as the unit of analysis (i.e., the ‘n’ of the test is always the number of subjects).

### Data availability

The authors declare that the data gathered for this study are available within the paper. We also state that the data are available from the authors upon reasonable request.

## Electronic supplementary material

Supplementary Information
