# Supplementary material for: Pupil Size Tracks Attentional Performance In Attention-Deficit/Hyperactivity Disorder
Source: Sci Rep. 2017 Aug 15;7:8228. doi: 10.1038/s41598-017-08246-w (PMC5557799; doi:10.1038/s41598-017-08246-w)
Supplement: Supplementary file 1 — Supplementary Information [file 41598_2017_8246_MOESM1_ESM.pdf]

TITLE: PUPIL SIZE TRACKS ATTENTIONAL PERFORMANCE IN ATTENTION-DEFICIT/HYPERACTIVITY DISORDER

Wainstein G[1], Rojas-Líbano D[1,2], Crossley NA[1,3], Carrasco X[4], Aboitiz F[1], Ossandón T[1*]

AFFILIATIONS:

1 Departamento de Psiquiatría, Escuela de Medicina and Centro Interdisciplinario de Neurociencia, Pontificia Universidad Católica de Chile, Santiago, Chile.

2 Laboratorio de Neurociencia Cognitiva y Social, Facultad de Psicología, Universidad Diego Portales, Santiago, Chile.

3 Department of Psychosis Studies, Institute of Psychiatry, Psychology and Neuroscience. King's College London, UK.

4 Servicio de Neurología y Psiquiatría, Hospital de Niños Dr. Luis Calvo Mackenna, Facultad de Medicina, Universidad de Chile, Santiago, Chile.

CORRESPONDING AUTHOR:

Tomás Ossandón

tossandonv@uc.cl

Department of Psychiatry - Medical School, Pontificia Universidad Católica de Chile. Marcoleta 381, Santiago, Chile

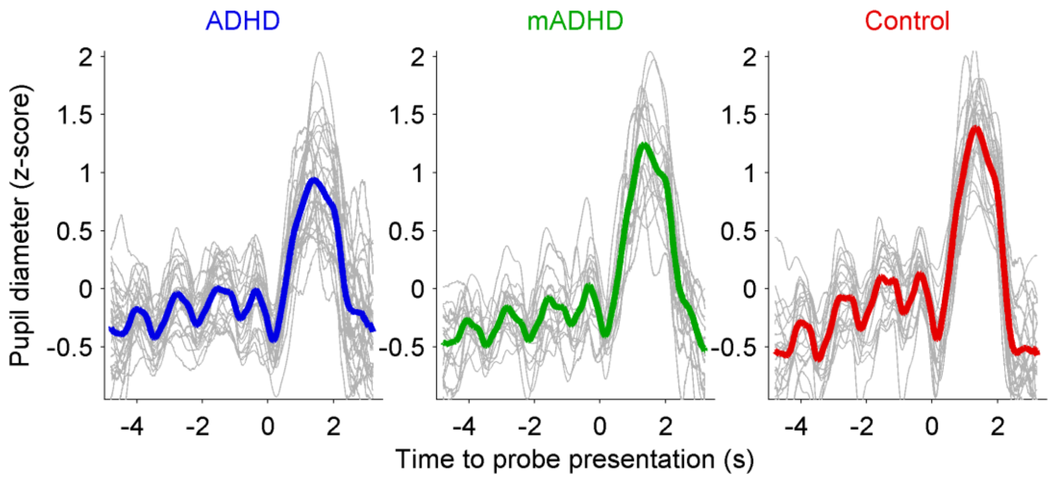

Supplementary Figure S1: Mean pupil change during a trial, separately by group. Each grey trace corresponds to the average pupil diameter from each subject (ADHD, n = 28; mADHD, n = 17; Control, n = 22). Color lines correspond to the mean group curve. Left: off-medication ADHD group; middle: on-medication ADHD group; right: Control group.

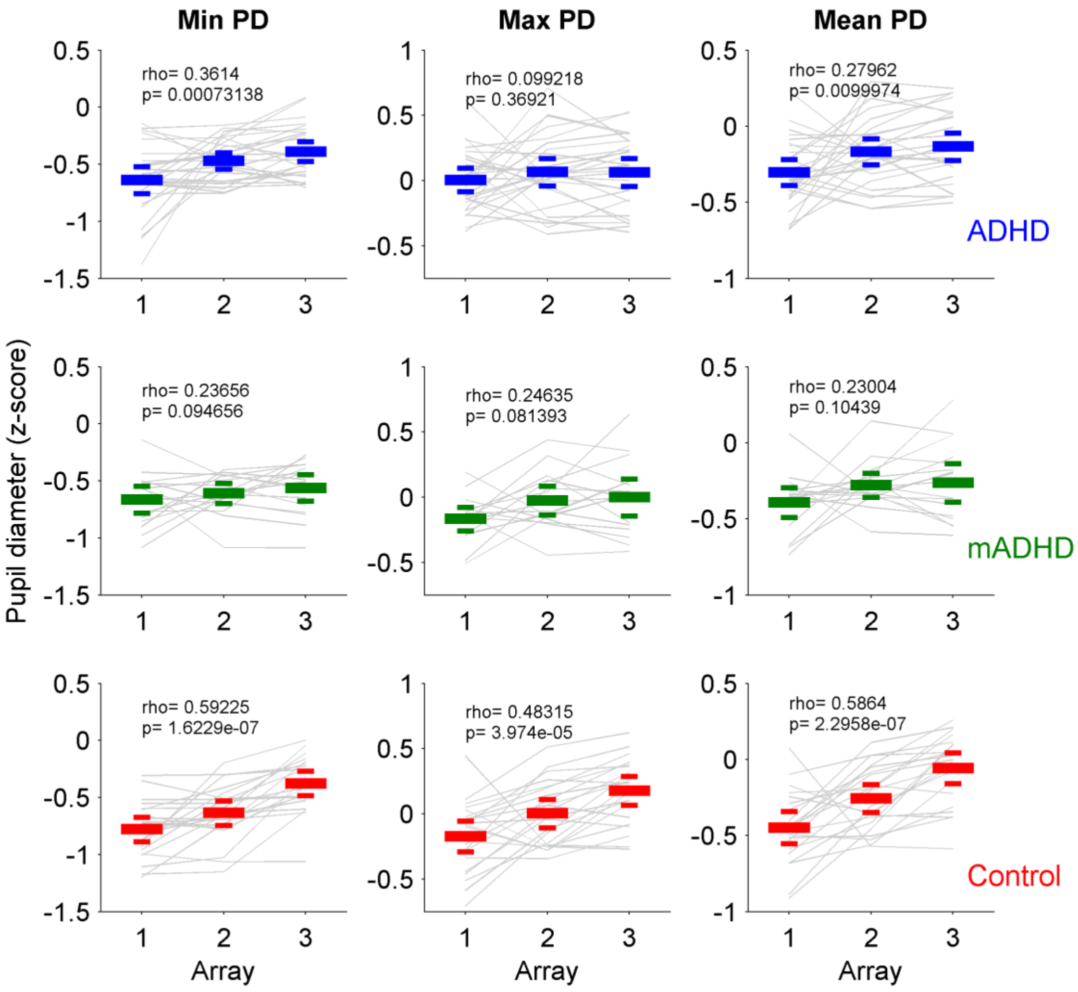

Supplementary Figure S2: Pupil diameter average magnitudes for the first three array presentations (see Figure 1A in the main text). Each grey trace corresponds to the session averages of each subject. Horizontal colored lines are the mean for each array across each group and the corresponding 95% confidence intervals. The values shown are the minimum pupil diameter (left column); maximum pupil diameter (middle column); and mean pupil diameter (right column). Different rows correspond to each group: first row is ADHD off-medication (blue color); second row is ADHD with medication (green color); and third row is control group (red color). The values of the Spearman correlation coefficient (r: rho), separately by group and magnitude, are shown at the top of each plot.

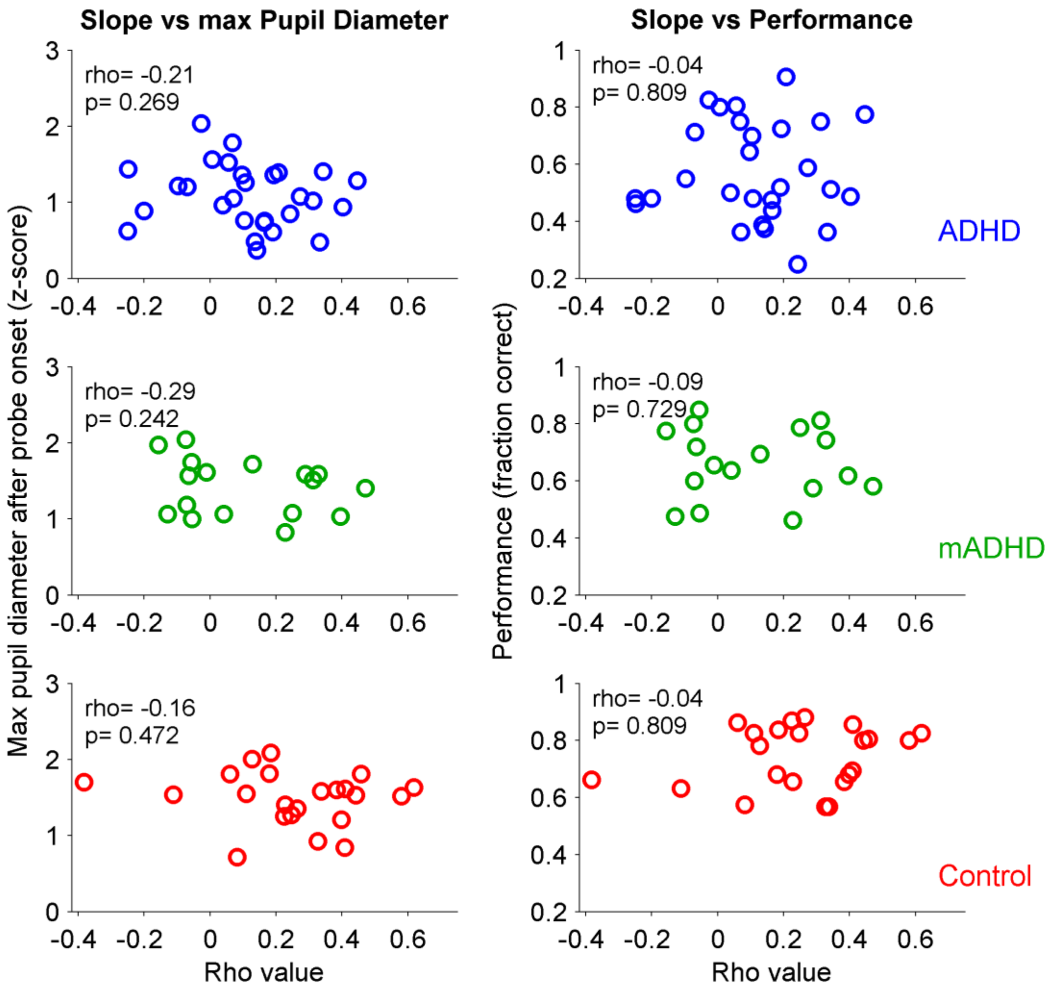

Supplementary Figure S3: Correlation between maximum pupil diameter after probe onset, performance and the pupil diameter slope of the first three arrays. Each circle corresponds to each subject's slope value, calculated as the Spearman correlation across the mean pupil diameter between the first three arrays (as shown in Supplementary fig. 2). Each of these values was plotted against the maximum pupil diameter after probe onset (left-side plots) and performance (right-side plots). Additionally, the rho value of the Spearman correlation is shown at the top of each plot.

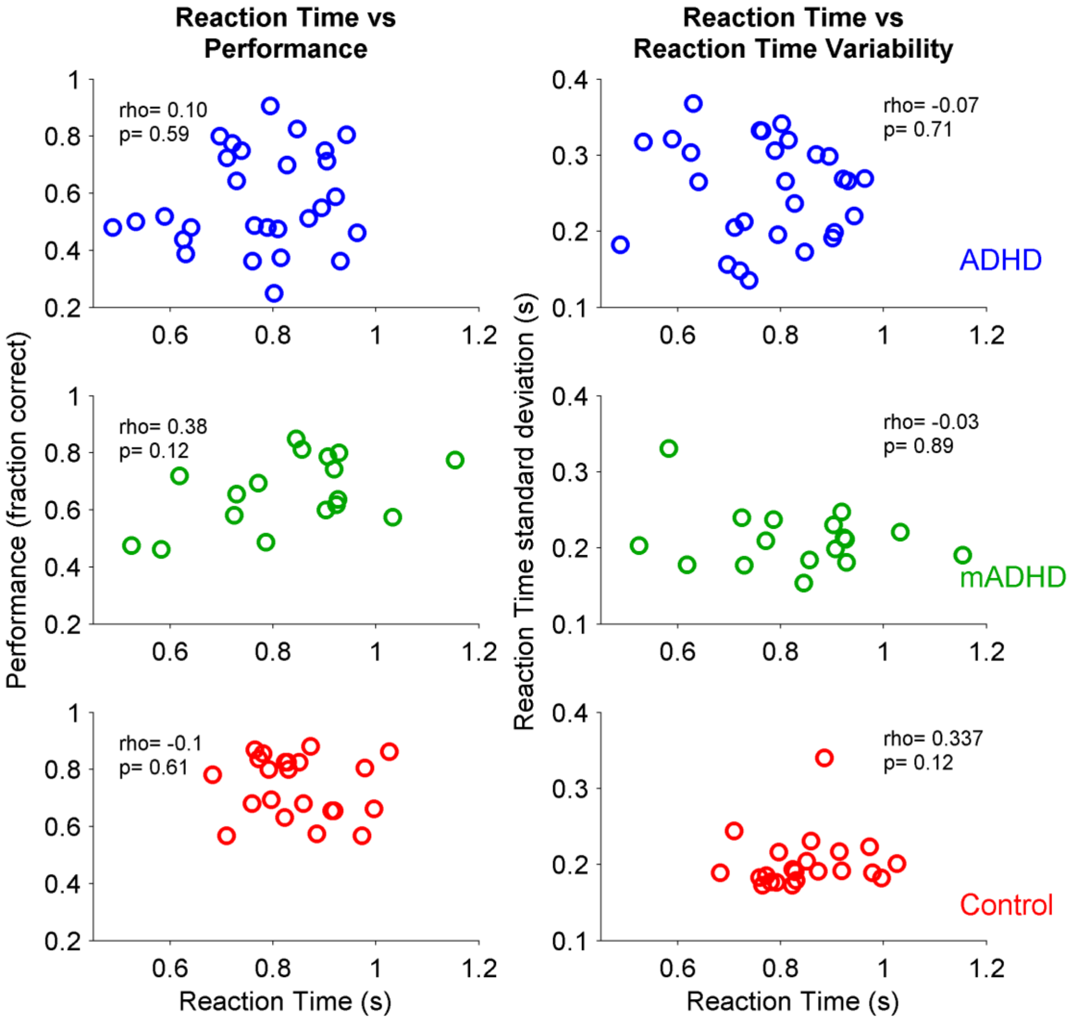

Supplementary Figure S4: Correlation between reaction time, performance and reaction time variability. Each dot corresponds to the subject's mean session value. The values of the Spearman correlation coefficient (r: rho), separately by group, are shown at the top of each plot.

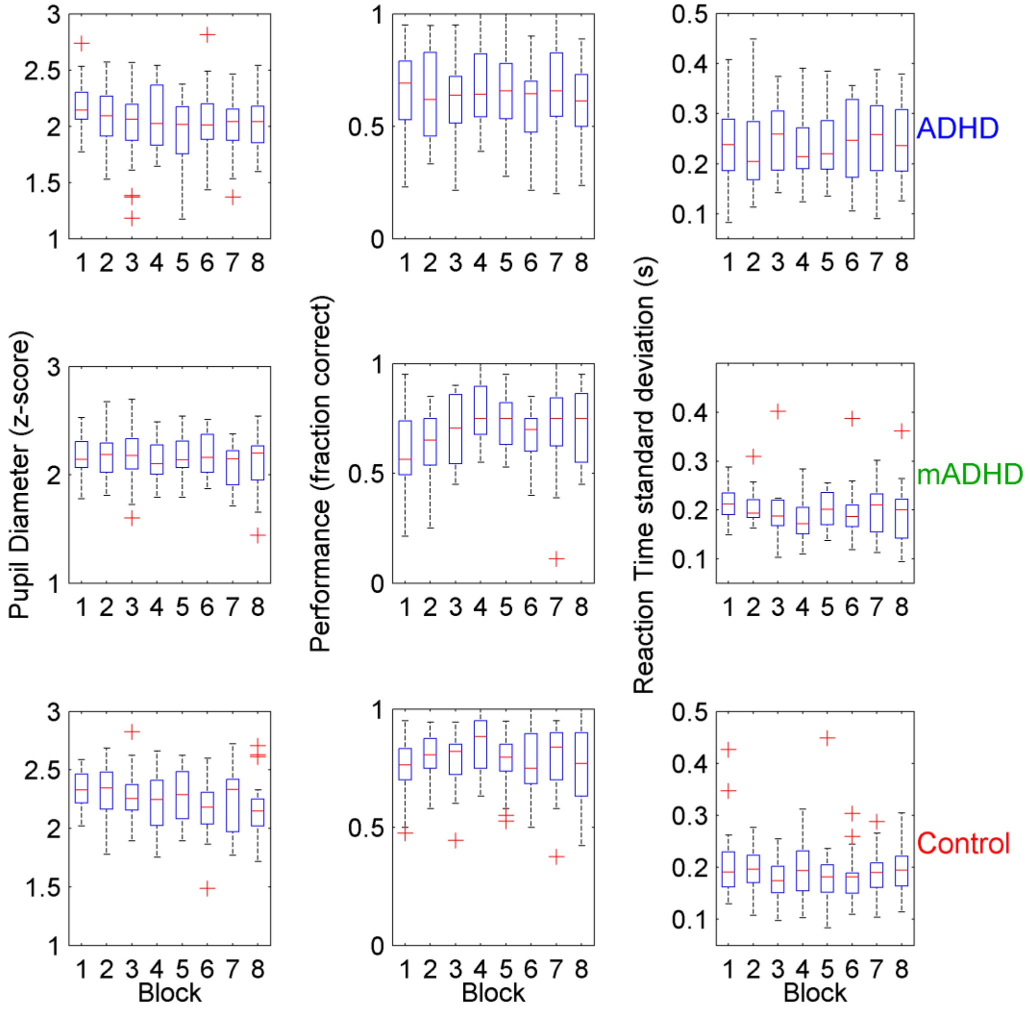

Supplementary Figure S5: Change of the maximum pupil diameter, performance and reaction time variability across the eight blocks of the task, for each group. Values are shown as boxplot for each group. Top three plots are the ADHD group; middle three plots are mADHD group; bottom plots are the control group. Right column plots are shown the maximum pupil diameter (z-score); middle column plots are the performance (fraction of correct responses); right column plots are the reaction time standard deviation.

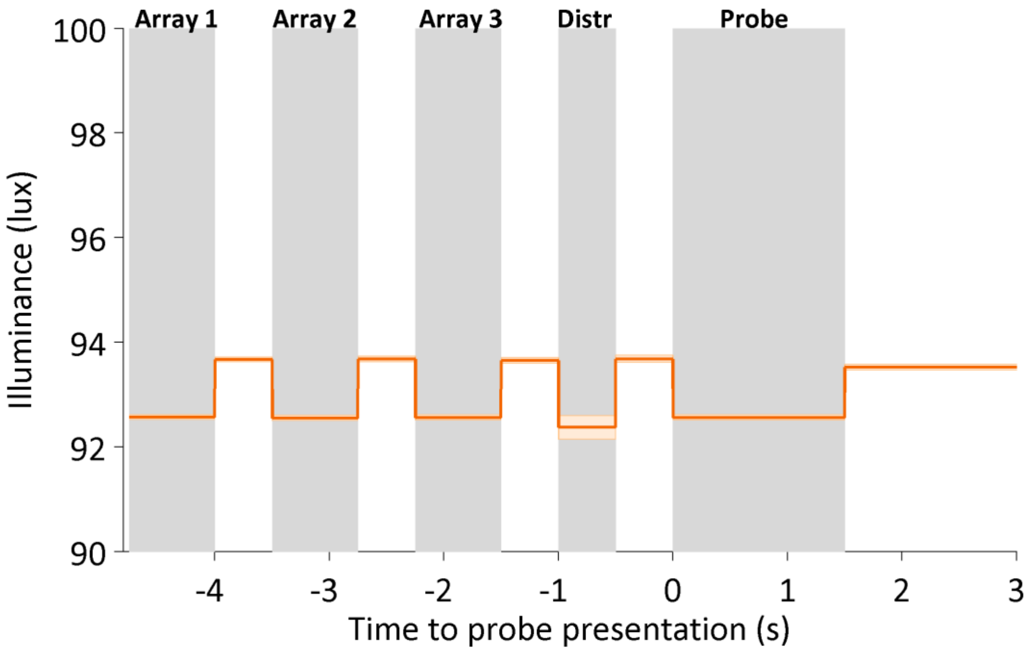

Supplementary Figure S6: Illuminance changes associated with the screen image changes during a trial. Grey shaded areas mark the periods of stimuli presentation. The thick orange line corresponds to the mean illuminance changes. Orange shaded areas correspond to the standard deviation of 42 measurement repetitions.
